# Investigations of Structural and Electrical Properties of ALD Films Formed with the Ozone Precursor

**DOI:** 10.3390/ma14185395

**Published:** 2021-09-18

**Authors:** Aleksandra Seweryn, Krystyna Lawniczak-Jablonska, Piotr Kuzmiuk, Sylwia Gieraltowska, Marek Godlewski, Robert Mroczynski

**Affiliations:** 1 Institute of Physics, Polish Academy of Sciences, Aleja Lotnikow 32/46, PL-02668 Warsaw, Poland; jablo@ifpan.edu.pl (K.L.-J.); kuzmiuk@ifpan.edu.pl (P.K.); sgieral@ifpan.edu.pl (S.G.); godlew@ifpan.edu.pl (M.G.); 2 Institute of Microelectronics and Optoelectronics, Warsaw University of Technology, Koszykowa 75, PL-00662 Warsaw, Poland; robert.mroczynski@pw.edu.pl

**Keywords:** ALD, AFM, MIS, high-*k* dielectric

## Abstract

The continuous development of ALD thin films demands ongoing improvements and changes toward fabricating materials with tailored properties that are suitable for different practical applications. Ozone has been recently established as a precursor, with distinct advantages over the alternative oxidizing precursors in the ALDs of advanced dielectric films. This study reports alumina (Al_2_O_3_) and hafnia (HfO_2_) formation using an O_3_ source and compares the obtained structural and electrical properties. The performed structural examinations of ozone-based materials proved homogenous high-*k* films with less vacancy levels compared to water-based films. The enhanced structural properties also result in the problematic incorporation of different dopants through the bulk layer. Furthermore, analysis of electrical characteristics of the MIS structures with ALD gate dielectrics demonstrated the improved quality and good insulating properties of ozone-based films. However, further optimization of the ALD technique with ozone is needed as a relatively low relative permittivity characterizes the ultra-thin films.

## 1. Introduction

Over the last two decades, atomic layer deposition (ALD) has been widely used in research and industry as an ideal technology for thin-film fabrication. ALD has a variety of advantages over conventional chemical vapor deposition (CVD) processes. Due to the unique self-limiting surface chemical reactions, ALD enables precise control of the thickness, composition, and stoichiometry of a grown material that is challenging to achieve via CVD methods [1,2]. Another essential advantage of ALD processing is its relatively low growth temperature, ranging from a typical 100 °C up to 350 °C. This feature makes ALD suitable for use in electronic and photonic devices based on transparent and flexible substrates, including polymers or hybrid structures with organic or low-dimensional materials [3,4,5]. Furthermore, ALD is commonly used for the fabrication of high-*k* dielectrics, transparent conductive oxides (TCOs), or conductive materials as gate-dielectrics in metal-oxide-semiconductor (MOS) devices [6,7], materials for non-volatile memory (NVSM) structures [8,9], optical coatings in lasers or light-emitting devices (LEDs) [10,11], membranes [12], sensing devices [13], waveguides [14], or photovoltaics [15,16].

The formation of thin films using ALD requires a precursor, efficiently supplying chemical reactions at the surface with reactive oxygen. Typical precursors that satisfy this requirement include oxygen (O_2_), hydrogen peroxide (H_2_O_2_), water (H_2_O), or ozone (O_3_), but water is most commonly used as the oxygen source during the fabrication of thin films. However, there are several reports that water-based ALD materials suffer from specific failures. It has been demonstrated that several films can delaminate from the substrate after elevated temperature treatment, and the substitution of water with ozone can limit such an effect [17,18].The growth of ozone-based materials can also be performed at significantly lower temperatures [19].

Furthermore, the improved quality of ALD materials has been shown to result in a significant smoothness level, lower leakage currents, and minor flat-band shift [20]. Ozone is characterized by a high electrochemical potential and high volatility. These two features are used in shortening purge times between ALD cycles and result in cost-savings [21]. The absence of hydrogen in the ozone molecule also reduces the risk of hydrogen and hydroxyl contamination in the deposited film.

Although the ALD method has been known for many years, and the first reports were from the 1970s [22,23], this method is still developing, which guarantees progress in thin films nanotechnology. In the case of oxide layers, the choice of the oxygen precursor can determine the properties of the layers. As we have already shown [24], the change from commonly used water to ozone significantly influenced, for example, the crystallography of ZnO layers. Selected metal precursors do not react with water due to the low activation energy. However, synthesis with ozone results in excellent film quality [25]. To systematize this issue after successful tests with dimethylzinc, experiments with another metal precursor were performed to examine if the properties of dielectric high-*k* oxide growth at low temperatures can be improved. In addition, to work with ALD ozone as a new oxygen precursor, the ALD procedures of high-k oxide growth using low temperatures, but with water, were developed [5].

In this work, we report the successful fabrication of alumina (Al_2_O_3_) and hafnia (HfO_2_) using an O_3_ source and compare the obtained structural and electrical properties of films. In the first stage of this study, we compare the structural and electrical parameters of Al_2_O_3_-based materials fabricated with water and ozone. Then, the selected processes were employed to fabricate alumina and hafnia layers with different thicknesses using an O_3_ oxygen source. The crystal structure, surface roughness, and chemical composition of fabricated films were analyzed. Moreover, the fabricated films were used as gate dielectric materials in MIS structures. Capacitance-voltage (C-V) and current-voltage (I-V) characteristics were examined to extract basic electrical parameters of investigated films. Our research study leads to the fabrication of good quality high-*k* films with properties suitable for electronic and photovoltaic applications.

## 2. Materials and Methods

### 2.1. ALD of High-k Oxides

**Figure 1:**
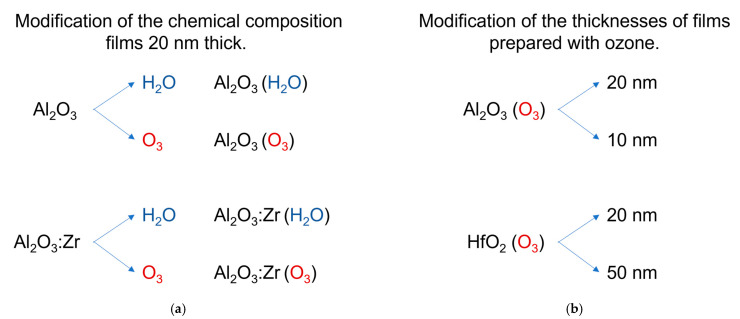
List of the samples investigated in this work: (**a**) modification of the chemical composition of films, (**b**) modification of the thicknesses of films.

ALD technology consists of the sequential deposition of metal and oxide monolayers, which result in the growth of oxide films. To grow alumina and hafnia thin films, we used the Savannah-100 Cambridge NanoTech Inc., Cambridge, Massachusetts, USA ALD reactor with access to the purging gas—nitrogen, with the purity of 6.0. This reactor was equipped with a Savannah Ozone Generator with access to oxygen with a purity of 5.0, as reported in [24]. According to the manufacturer’s data, the used ozone generator was characterized by ~120 mg/L (~7.5 wt.% O_3_) capacity. We performed the growth of films at low temperatures, i.e., 100 °C and 90 °C for alumina and hafnia deposition, respectively. As metal precursors, we used the following compounds: trimethylaluminum (CAS Number: 75-24-1, Sigma Aldrich, Saint Louis, MO, USA), tetrakis(dimethylamido)hafnium(IV) (CAS Number: 19782-68-4, Sigma Aldrich, Saint Louis, MO, USA) for the growth of films, and tetrakis(dimethylamido)zirconium(IV) (CAS Number: 19756-04-8, Sigma Aldrich, Saint Louis, MO, USA) as dopant compounds for the preparation of the alumina film with the zirconium ions. According to the type of films, the used oxidants were deionized water (Millipore Milli-Q) or ozone received from the generator mentioned above. As previously reported, all processes were performed under vacuum, below 0.5 Torr [24,26]. Several ALD cycles were optimized to deposition of 10 nm and 20 nm films thick in the case of alumina and 20 nm and 50 nm films thick in the case of hafnia. Moreover, the modification of films was done by selecting oxygen precursors and through doping of Zr ions. In Figure 1, all types of investigated samples are summarized. The growth rate by ozone-based ALD processes is comparable to that with water-based oxygen precursors. To obtain 20 nm thick Al_2_O_3_ film with water 128 ALD cycles are needed up to 150 ALD cycles to get 20 nm thick Al_2_O_3_ film with water. This gives a growth rate of 1.6 Å/cycle and 1.3 Å/cycle for alumina water and ozone-based ALD appropriately. The result obtained for Al_2_O_3_:Zr were 1.5 Å/cycle and 1.2 Å/cycle with water and with ozone, respectively. The growth rate using hafnia ALD growth with the ozone was 1.4 Å/cycle.

### 2.2. Structural Characterization of Dielectric Films

In order to describe the chemical composition and structural properties of films, selected measurements were performed. The film thicknesses were defined using a NanoCalc 2000-UV/VIS (Micropack GmbH, Ostfildern, Germany) with pre-installed software(Nanocalc232, Ostfildern, Germany).

XPS measurements were performed with a Scienta (Uppsala, Sweden) R4000 hemispherical analyzer (pass energy 200 eV) and monochromatic Al K_α_ (1486.7 eV) excitation (Scienta MX-650) working with a power of 150 W. The full width at half maximum (FWHM) of the 4f7/2 Au line measured under the same experimental conditions was 0.64 eV. The energy scale was calibrated, setting the C 1s line at the position of 285.0 eV. Samples were measured as received. A significant amount of C was detected on the sample surface. To gain information about the content of C in the bulk of the sample, short sputtering with an Ar^+^ ion gun at 1 kV, 5 mA, was performed. The spectra were analyzed using the commercial CASA XPS software package (Casa Software Ltd., version 2.3.17) with the Shirley background. The spectra were fitted with a mixed Gaussian–Lorentzian (GL (30)) function.

The surface morphology was investigated using atomic force microscopy (AFM, Bruker Dimension Icon, Santa Barbara, CA, USA) using PeakForce Tapping and silicon nitride probes with sharp tips (tip radius—2 nm). The surface roughness was determined by a root mean square (RMS) roughness of the AFM height measurements from images taken from a 10 × 10 μm^2^ region. The surface morphology of the layer was measured on silicon substrates.

### 2.3. MIS Structures Fabrication and Electrical Characterization

To perform electrical characterization of examined high-*k* films, MIS capacitors with Al_2_O_3_ or HfO_2_ gate dielectric layer were fabricated. In this study, silicon (Si) n-type substrates with a resistivity of 1 ÷ 10 Ωcm and an orientation (100) were used. The processing sequence of the MIS structures was as follows: Si substrates were cleaned employing a modified RCA (Radio Corporation of America) method (Piranha + SC1 + SC2 + HF dipping). Then, ALD processes, according to the procedure described in Section 2.1 were performed. After forming ALD oxides, the aluminum (Al) contact pads were developed through the standard UV (@400 nm) photolithography process and wet etching of excess aluminum. A pulsed-DC magnetron sputtering process was used to fabricate conductive films [27]. A PlasmaLab Oxford System 400 (Bristol, UK) was used to perform Al deposition on the top and bottom contacts. The fabricated MIS structures with the gate area of A = 1.7 × 10^−4^ cm^2^ allowed for determining the investigated structures’ basic electrical properties. The quality of obtained MOS structures was examined with a Keithley 4200 semiconductor characterization system (Tektronix, Beaverton, OR, USA) equipped with SUSS PM-8 probe station utilizing current–voltage (I-V) and capacitance–voltage (C-V) characteristic analyses. The procedure of the extraction of electrical parameters of the examined MIS structures was described in [28].

## 3. Results and Discussion

### 3.1. Structural Characterization of Investigated Samples

The uniform and homogenous ALD hafnia and alumina films were obtained on the Si substrate. First, the growth rates were estimated by performing ALD processes to define the number of ALD cycles needed. The thickness of the layers was in the range of 20–22 nm. This confirms the repeatability and scalability of the ALD measurement, which is one of the main advantages of this technology. The amorphous nature of the structures grown at low temperature has been shown in previous work [5].

Table 1 presents elements content (in at %) in investigated samples derived from XPS measurements of C 1s, O 1s, Al 2p, Hf 4f and Zr 3d lines.

**Figure 2:**
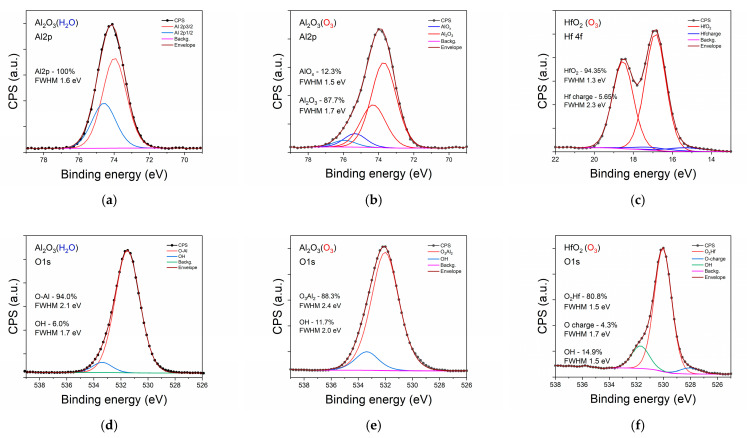
High-resolution spectra for metals and oxygen for investigated samples of pure Al_2_O_3_ and HfO_2_ as measured for bulk, after short Ar^+^ sputtering to remove surface contamination Al line: (**a**–**c**), O line (**d**–**f**).

**Figure 3:**
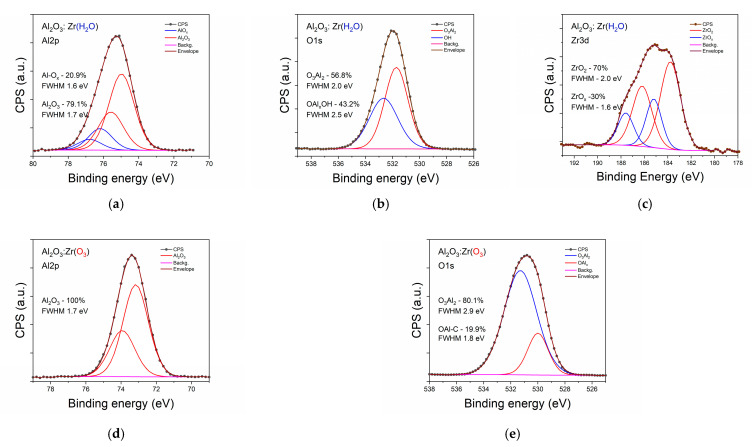
High-resolution spectra for metals and oxygen for investigated samples of pure Al_2_O_3_ doped with Zr ions as measured for bulk, after short Ar^+^ sputtering to remove surface contamination: with water (**a**–**c**), with ozone (**d**,**e**).

Considering the content of the component in the investigated samples from the data presented in Table 1, is clearly seen that only the bulk of the HfO_2_ sample is stoichiometric, but, at the surface of this sample, an excess of oxygen was detected together with a high content of C. Opposite to this, in the case of Al_2_O_3_ samples, a deficit of oxygen was found in all samples besides that of Al_2_O_3_:Zr (O_3_). The growth performed in the presence of ozone reduced the number of oxygen defects in the Al_2_O_3_ (Al/O = 0.70 compared to 0.77 in the case of Al_2_O_3_ (H_2_O)) but prevented the incorporation of Zr. This suggests that Zr diffused through the oxygen vacancies during sample growth. The oxygen vacancies at the level of 0.77 were naturally present in the crystalline sapphire and significantly affected the chemistry of N incorporation from the nitrogen plasma [29]. The presence of O vacancies was necessary for the incorporation of N. Moreover, the increase in the number of O vacancies by forming the Al(NO_y_)_x_ in the amorphous AlO_x_ films significantly increased the amount of incorporated N. To check this hypothesis, an analysis of chemical states of the measured high-resolution spectra was performed. The results are presented in Figure 2 and Figure 3. For clarity of presentation, only the data for the bulk of the samples is shown. Examining the Al 2p spin-orbital in two cases (Al_2_O_3_ (H_2_O) and Al_2_O_3_:Zr (O_3_)), single chemical binding of Al was detected at the surface (not shown) and in the bulk of the samples with slightly better chemical order in the first sample (full width at half maximum (FWHM) 1.6 eV and 1.7 eV, respectively).

Nevertheless, the oxygen 1s orbitals for these samples had different components, indicating the different chemistry of oxygen incorporation. In the case of Al_2_O_3_ (H_2_O) 94% of the oxygen had binding energy (BE) for sapphire (Figure 2d). Other atoms were shifted more than 1.5 eV to higher energy, typical for absorbed water or other contamination [30]. For the Al_2_O_3_:Zr (O_3_) sample, two oxygen components provided a good envelope for a measured line, but the second component that took 20% of O had a BE lower by 1.3 eV compared with the fraction banded in sapphire. Moreover, the mainline was much broader (FWHM 2.9 eV) than in Al_2_O_3_ (H_2_O) (FWHM 2.1 eV). This low BE fraction consisted at the surface of more than 50% of the oxygen. This kind of fraction was not observed in the case of Al_2_O_3_ (O_3_) without Zr doping. In addition, Zr atoms, neither in bulk nor at the surface of Al_2_O_3_:Zr (O_3_), were detected. This may suggest that the low energy oxygen fraction filed the oxygen vacancies in the Al_2_O_3_ crystalline structure and formed higher valency component [31] and blocked Zr incorporation.

Analyzing the chemistry observed in the Al_2_O_3_:Zr (H_2_O) sample, where Zr was detected, at a level of 1.3 at % at the surface and 2.2 at % in bulk, we can clearly distinguish two fractions of Al and Zr as well at the surface, as in bulk. This fact demonstrates the presence of the fraction of Al atoms with a distorted atomic order compared to that in crystalline sapphire. At the surface, it is about 42% Al atoms and in bulk 21%, indicating that the process begins at the surface and advances to the bulk of the film. The second component has a higher BE, which is usually connected with fewer oxygen atom bonds to metal (e.g., [32] for MnO_2_ BE of Mn(IV) is 641.4 eV but fir MnO 641.1 eV Mn(II)) and a higher ionic state. In the case of Zr at the surface, only 50% of all Zr atoms have atomic order as in ZrO_2_ while 70% do in bulk. Two components of the O 1s line provide good fitting, one with BE for sapphire and a high BE component evidencing other kinds of oxygen bindings. High energy components at the surface consist of almost 79% O. Still, in bulk, only 43% is much broader than the sapphire component, which usually shows several different atoms surrounding in this fraction.

Two kinds of Al bonds were also detected in the case of the Al_2_O_3_ (O_3_) sample, confirming the lack of oxygen, but the second higher BE fraction was at the level of 10% (20% in the case of the Al_2_O_3_:Zr (H_2_O) sample). The second fraction of oxygen was also much smaller in the Al_2_O_3_ (O_3_) sample. For HfO_2_ (O_3_), the one fraction of Hf 4f was used to fit the spectra and two typical components for O 1s (Figure 2c, f), but a small charging effect was observed at the low energy site of both spectra.

Summing up, XPS studies showed that films grown in the presence of ozone have fever oxygen vacancies than those grown in the presence of H_2_O, but this prevents the accommodation of dopants.

**Figure 4:**
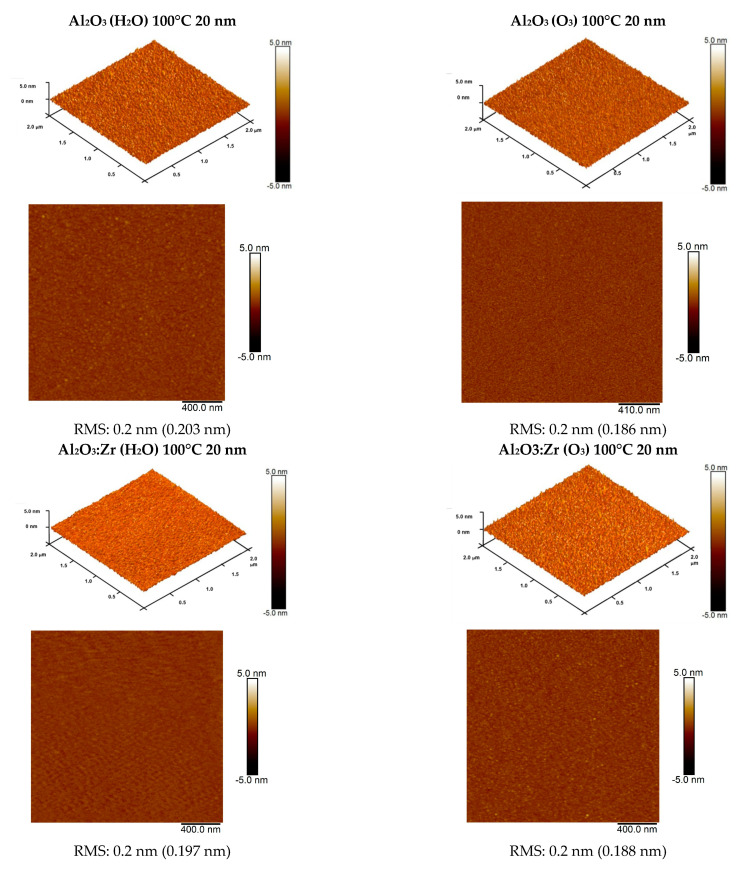
AFM images (2 × 2 μm^2^) and RMS roughness values of Al_2_O_3_ and Al_2_O_3_:Zr thin films (~20 nm thick) grown with ALD from water (H_2_O) or ozone (O_3_) at 100 °C on Si substrate.

**Figure 5:**
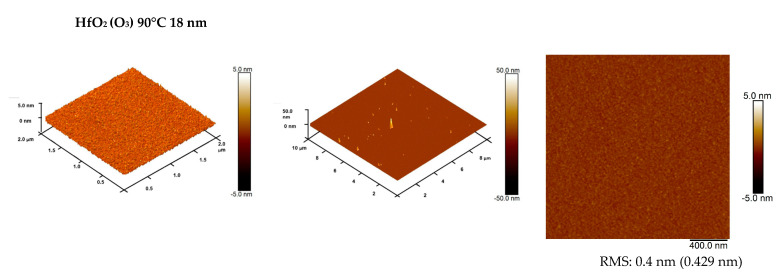
AFM images (2 × 2 μm^2^ and 10 × 10 μm^2^) and RMS roughness value of HfO_2_ thin films (20 nm thick) grown with ALD from ozone (O_3_) at 90 °C on Si substrate.

Since the low roughness of the surface is a crucial parameter for the electrical properties of the films, AFM measurements were performed. The data are presented below. Figure 4 and Figure 5 indicate a very flat surface for low-temperature thin films grown using ALD with a thickness of approximately 20 nm. The extremely low surface roughness with an RMS roughness value of about 0.2 nm was achieved for all aluminum oxide layers, including Al_2_O_3_ and Al_2_O_3_ with Zr obtained at 100 °C from water or ozone as the oxide precursor. It can also be clearly noticed that a slightly better surface morphology can characterize high-*k* films fabricated using O_3_. HfO_2_ layers deposited at 90 °C are characterized by a somewhat higher surface roughness, but are still very homogenous, with an RMS roughness level of about 0.4 nm.

The processes of ALD deposition for the HfO_2_ and Al_2_O_3_ layers were investigated by the authors [5,33]. The Hf and Al precursors used by us in the present paper are the same—tetrakis(dimethylamido)hafnium (TDMAH) and trimethylaluminum (TMA), respectively—as in our previous studies. The oxygen precursor used in previous experiments was deionized water:

Hf [(CH_3_)_2_N]_4_ + 2H_2_O → HfO_2_ + 4HN(CH_3_)_2_

2Al(CH_3_)_3_ + 3H_2_O → Al_2_O_3_ + 6CH_4_

where ozone is used in the present paper. We determined the so-called ALD growth window from earlier studies. We established that the ALD window for HfO_2_ is in the range of 130 °C to 140 °C, giving the growth rate of 1.4 Å per cycle, and, for Al_2_O_3_, from 180 °C to 200 °C, giving a growth rate of 1.0 Å per cycle. However, both films can be deposited at lower temperatures. At 80 °C, the growth rate for Al_2_O_3_ drops slightly to 0.8 Å per cycle [33]. A reverse effect was observed for HfO_2_, i.e., the growth rate was even higher, about 1.5 Å per cycle [33]. For HfO_2_, we observed that films with a thickness above about 200 nm showed a tendency to crystallize, even at low process temperatures. For Al_2_O_3_ layers, this tendency is weaker (as we concluded from XRD investigations) [33], but is still a lower growth temperature and thinner films are more suitable for microelectronic applications. This is because the amorphous structure is expected to have substantially reduced leakage current. Higher leakage currents are associated with the presence of grain boundaries appearing in crystalline films [2]. We also observed that films grown at a lower temperature are very smooth. From the AFM studies, we concluded that the surface roughness’s root mean square (RMS) value depends on the growth temperature and the layer thickness. It was the lowest (RMS = 0.2 nm) for the thin Al_2_O_3_ films (50 nm) and a growth temperature of 100°C [33]. Based on the results discussed above, we selected a low growth temperature for both Al_2_O_3_ and HfO_2_ layers but changed the source of oxygen from deionized water to ozone. From initial studies, we checked that such changes affected the material properties and may affect hydrogen and carbon concentrations in the films and film stoichiometry. Regarding film stoichiometry, since ozone is more reactive, we expected that the concentration of vacancies in the films might be affected.

### 3.2. Electrical Characterization of MIS Structures with ALD Films as Gate-Dielectric Layers

**Figure 6:**
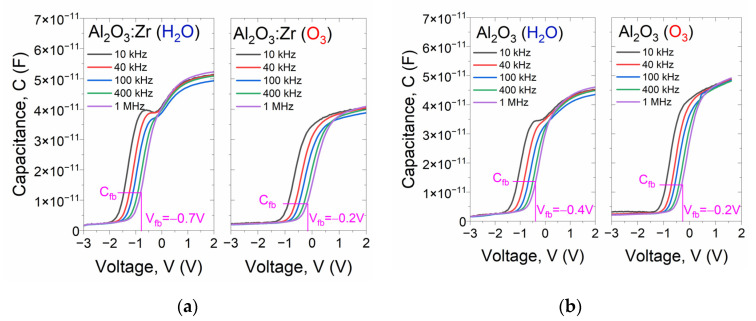
The typical family of high-frequency C-V characteristics of MIS structure with 20 nm ALD gate dielectric film investigated in this work: (**a**) Al_2_O_3_ and (**b**) Al_2_O_3_:Zr.

The analysis of electrical parameters of high-*k* films was started with a comparison of MIS structures with Al_2_O_3_-based gate dielectrics fabricated using H_2_O and O_3_ precursors. At first, MIS devices with 20 nm Al_2_O_3_ and Al_2_O_3_:Zr films were fabricated. In Figure 6, a comparison of the family of C-V characteristics of MIS capacitors is presented.

**Figure 7:**
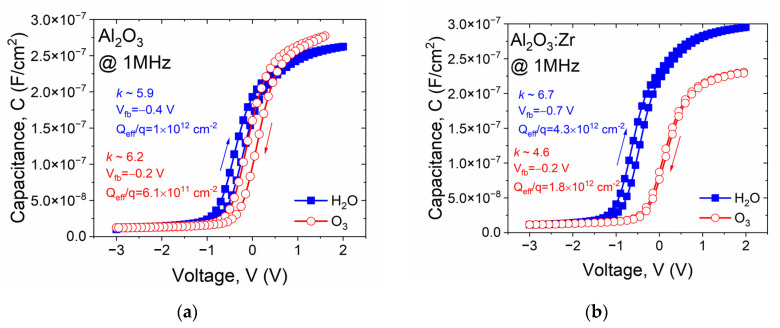
Comparison of the hysteresis loop of representative C-V characteristics (signal frequency, f = 1 MHz) of MIS structures with Al_2_O_3_-based gate dielectric layer: (**a**) Al_2_O_3_ and (**b**) Al_2_O_3_:Zr.

It is worth noting that the C-V characteristics of the structures with Al_2_O_3_-based materials using H_2_O can be characterized using a characteristic “knee” in the depletion regime of silicon. The magnitude of this effect is more prominent as the signal frequency becomes lower. This implicates the presence of charge traps in the dielectric film, i.e., shallow traps that keep up with the signal frequency. This effect does not occur in ozone-based films, suggesting a more effective saturation of dangling bonds during the ALD process employing the O_3_. Moreover, the flat-band (V_fb_) voltage values of the C-Vs of MIS devices with ozone-based films are smaller (in absolute values) than in ALD films fabricated with a water precursor. This is related to the generation of a positive charge in the dielectric layer bulk [34]. One of the possible mechanisms of the positive charge formation is the generation of hole trap sites induced by broken bonds at the semiconductor/dielectric charge traps that are significantly large. Thus, the dielectric quality of high-*k* films fabricated with O_3_ is improved, which was further verified by comparing C-Vs interfaces. These traps are formed during the growth of a dielectric film. As the ozone precursor is more chemically reactant than water, water-based dielectric films (@1MHz) are depicted in Figure 7.

The results presented in Figure 7 confirm that Al_2_O_3_ fabricated with an ozone precursor is characterized by a higher relative permittivity, lower V_fb_ (in absolute values), and lower effective charge (Q_eff_/q) compared to a material formed using H_2_O precursor. The magnitude of a hysteresis loop is comparable for both films. The comparison of C-Vs of MIS devices with Al_2_O_3_:Zr films also revealed better electrical parameters for the high-*k* film formed with O_3_, which manifests in a similar improvement of dielectric quality material, even with the disappearance of the C-V hysteresis loop. However, a relatively lower relative permittivity was obtained, i.e., 6.7 and 4.6 for H_2_O-based, and O_3_-based ALD film, respectively.

**Figure 8:**
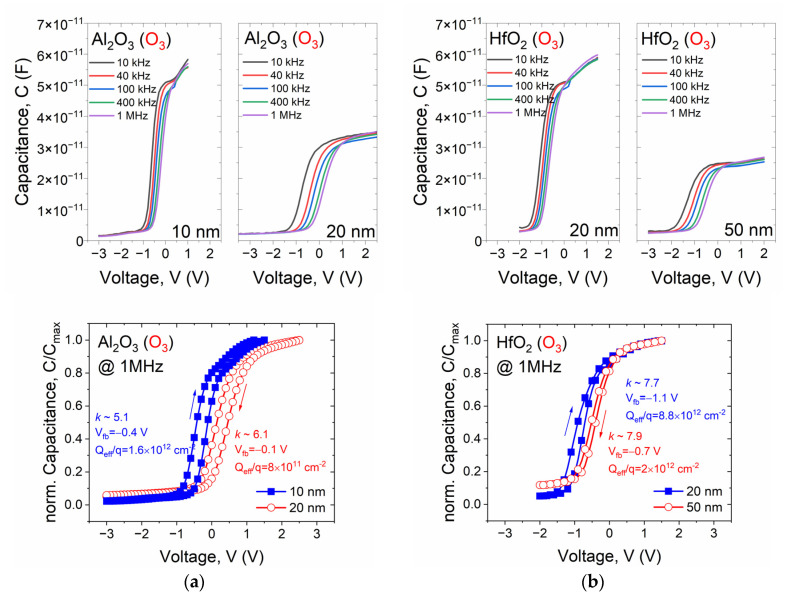
Comparison of representative high-frequency C-V characteristics of MIS structures with ALD gate dielectric films deposited with O_3_ precursor: (**a**) Al_2_O_3_ and (**b**) HfO_2_.

MIS structures with the ALD Al_2_O_3_ and HfO_2_ O_3_-based layers with different thicknesses were fabricated in the next step. In the case of aluminum oxide, 10 nm- and 20 nm-thick materials have been deposited, while in the case of hafnium oxide—20 nm and 50 nm. In Figure 8, a comparison of the families of C-V characteristics of the examined materials is presented. Moreover, the magnitude of hysteresis loops of both types of MIS stacks is also depicted. As the different thicknesses characterize the considered materials, the depicted C-Vs are constructed as normalized capacitance levels to each stack’s maximum capacitance.

After analyzing the data presented in Figure 8, it can be concluded that the frequency dispersion is smaller than in thicker oxides in both cases for the MIS devices with thinner dielectric films. The presence of charge traps in the depletion region also becomes more noticeable, which is not observed in the case of investigated capacitors with thicker Al_2_O_3_ and HfO_2_ films. Furthermore, a slightly higher permittivity and lower V_fb_ values (in absolute values) have been demonstrated in both thicker films. These findings are further confirmed by a lower effective charge density, as shown in Figure 8.

It can also be noticed that a typically higher permittivity value and slightly higher Q_eff_/q values characterize the obtained hafnium oxide films compared to Al_2_O_3_. The latter observation may result from the fact that an inhomogeneous interfacial layer (IL) between the semiconductor and the hafnia film may have formed, which is a typical effect for hafnium-based dielectric material deposition on silicon [35]. It has already been shown that, due to the chemical instability of the hafnium oxide films, oxygen atoms easily diffuse towards the semiconductor/dielectric interface. Thus, a very thin IL is formed which deteriorates, not only the properties of the MIS stack, but also reduces the permittivity value of the dielectric film. Simultaneously, the flow of oxygen atoms from the dielectric layer bulk results in a deterioration of the stoichiometry of HfO_x_ film. The latter also contributes to lowering the electrical quality of the investigated dielectric stack.

**Figure 9:**
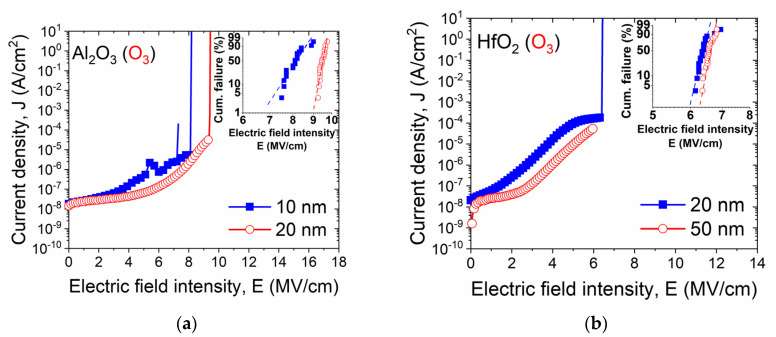
Comparison of representative I-V characteristics of MIS structures with ALD gate dielectric layers expressed as gate leakage current density vs. electric field intensity for (**a**) Al_2_O_3_ and (**b**) HfO_2_; Weibull plots of cumulative breakdown distribution vs. electric field intensity are also shown as insets.

Considering the insulating properties of ALD materials, the measured current–voltage (I-V) characteristics of fabricated MIS devices have been compared and expressed as gate leakage current density vs. electric field intensity (Figure 7). The presented data in Figure 9 compare the leakage current density of MIS stacks with investigated ALD films. As the inset, a cumulative failure analysis is depicted as Weibull plots. Around twenty MIS capacitors with each gate dielectric material were examined to introduce the statistical data of the distribution of breakdown voltage values.

The analyzed I-V characteristics of MIS devices with each type of O_3_-based ALD material showed very similar insulating properties in this study. However, a slightly lower leakage current density was observed for thicker ALD films, resulting from improved electrical properties described during C-Vs analysis. For Al_2_O_3_ films, up to ~6 MV/cm, a relatively low leakage current density, was demonstrated, i.e., between 10^−8^ and 10^−7^ A/cm^2^. Then, the leakage current increased and the breakdown occurred between 7 and 8 MV/cm, and 9 MV/cm, for thinner and thicker Al_2_O_3_ film, respectively. Similar results can be found in [36,37]. For instance, in [36], depending on the temperature of the ALD process, the breakdown phenomenon was observed at 7.0 and 6.3 MV/cm for Al_2_O_3_ deposited at room temperature and annealed at 400 °C, respectively. A significantly lower breakdown voltage value, i.e., 5.3 MV/cm, for a MIS stack with Al_2_O_3_ deposited at 177 °C was demonstrated in [37].

In the case of a MIS structure with a HfO_2_ film, the leakage current density is within the range of 10^−8^ and 10^−7^ A/cm^2^ up to 2 MV/cm, and then starts to increase, and the breakdown occurs at a relatively high electric field intensity, between 6 and 7 MV/cm. It is worth noting that for both investigated thicker high-*k* films, the distribution of breakdown voltage values is very narrow, i.e., around 90% of structures fail within the range of 0.5 MV/cm.

The results in the present work show very good electrical properties of the films grown with ozone. For example, for Al_2_O_3_ films, up to ~6 MV/cm leakage current density is between 10^−8^ and 10^−7^ A/cm^2^. Similar values are obtained for HfO_2_ films. A leakage current must be as small as 10^–7^ A/cm^2^ at 1 V for applications in non-volatile memories [38,39,40]. In addition, we examined if the small amount of Zr in Al_2_O_3_ films will increase the chance of use of Al_2_O_3_ application in Si-based microelectronics. Considering the required properties of alternative oxides to replace SiO_2_ as a gate [35,41,42], Al_2_O_3_ films are best but were not used since the dielectric constant is too low. This may be changed by introducing some amount of Zr, since the dielectric constant of ZrO_2_ is similar to that of HfO_2_. The first results presented in this study are encouraging and makes the investigated ozone-based ALD materials very promising for future practical applications. The dielectric materials studied in this work can be characterized by suitable electrical parameters for most applications in MOS/MIS semiconductor devices and other structures where dielectric material electrical performance is crucial. However, the obtained relatively lower permittivity noted that the deposition processes need further studies and optimization for MIS structures and devices applications. The considerably lower *k*-values compared to those commonly found in the literature may be due to the lower density of the obtained films and/or the interfacial layer and quantum mechanical effects [43]. As the results presented in this work prove the relatively high density of deposited films, we believe that the lower permittivity values can be attributed to the presence of IL film and the deteriorated interfacial properties reported in this work. The presence of an interfacial dielectric layer adds an extra capacitor in series with a high-*k* film that lowers the calculated permittivity value. The additional IL is formed from a silicon oxide (SiO_x_) material with a permittivity value of ~3.9 or lower. Simultaneously, non-stoichiometric IL also deteriorates the MIS stack’s electrical properties, as shown in the present work. Thus, optimizing ALD technology and finding the best growth parameters are of importance for the future.

## 4. Conclusions

The structural and electrical properties of ALD alumina and hafnia thin films have been investigated. The high-*k* materials were fabricated using water and ozone as the oxygen precursors. All films were homogenous with low RMS values, as AFM showed. The films grown with ozone are characterized by a smaller amount of oxygen vacancies compared to those grown with water, but this prevents the accommodation of dopants. The present study also indicated that an ozone oxygen precursor is superior to a water one, resulting in films with better electrical parameters. Such films show a low hysteresis of C-V characteristics, higher breakdown voltages, and lower leakage current, which is important for MIS structure applications. However, the technology still needs optimization and further investigations to fully understand the chemical reactions that occur during the growth of the high-*k* film towards improving the relative permittivity value.

## Figures and Tables

**Table 1:** Elements content (in at %) in investigated samples from XPS measurements of C 1s, O 1s and Al 2p, Hf 4f and Zr 3d lines. “Surface” is related to sample measure as received, “bulk” to sample after sputtering with Ar^+^ ions with 1 kV energy.

Sample	C_surface_	C_bulk_	O_surface_	O_bulk_	Al(Hf)_surface_	Al(Hf)_bulk_	Zr_surface_	Zr_bulk_
Al2O3 (H2O)	14.2	4.9	48.5	53.7	37.3 Al/O = 0.77	41.4 Al/O = 0.77		
Al2O3 (O3)	20.5	4.8	46.8	54.9	32.7 Al/O = 0.70	40.3 Al/O = 0.73		
Al2O3:Zr (H2O)	18.6	4.5	46.8	52.7	33.3 Al/O = 0.71	40.6 Al/O = 0.77	1.3	2.2
Al2O3:Zr (O3)	15.6	5.9	50.5	55.8	34.0 **Al/O = 0.67**	38.3 Al/O = 0.69		0
HfO2 (O3)	33.9	4.5	46.0	63.7	20.1 Hf/O = 0.44	31.8 **Hf/O = 0.5**		
